# A New Hæmostatic

**Published:** 1874-03

**Authors:** 


At the recent meeting of the Italian Scientific Congress, held in Rome, two Neapolitan physicians submitted for examination a liquid preparation designed for stopping instantaneously the flow of blood from wounds of every description. A commission of physicians, according to the Roman "Fanfulla," have been experimenting with it in the anatomical theatre of the Santo Spirito, and have reported on it as one of the happiest of recent discoveries, and as particularly serviceable on the field of battle.

